# Molecular cloning and defence functional analysis of *MaNPR11* from Cavendish banana (*Musa* spp.)

**DOI:** 10.3389/fpls.2026.1763303

**Published:** 2026-03-16

**Authors:** Jing-Yi Wang, Xia Wang, Cai-Hong Jia, Meng-Ling Zhu, Yan-Yun Lv, Zhuo Wang

**Affiliations:** 1 State Key Laboratory of Tropical Crop Breeding, Institute of Tropical Bioscience and Biotechnology & Sanya Research Institute, Chinese Academy of Tropical Agricultural Sciences, Haikou, China; 2 Hainan Key Laboratory for Protection and Utilization of Tropical Bioresources, Hainan Institute for Tropical Agricultural Resources, Chinese Academy of Tropical Agricultural Sciences, Haikou, China; 3 National Key Laboratory of Crop Genetic Improvement, Huazhong Agricultural University, Wuhan, China

**Keywords:** banana, disease resistance, *Fusarium oxysporum*, NPR1, systemic acquired resistance

## Abstract

Bananas (*Musa* spp.) are globally critical economic crops, but the commercially dominant Cavendish cultivars are highly susceptible to *Fusarium oxysporum* f. sp. *cubense* (Foc), a destructive soil-borne pathogen. It severely undermines the healthy and sustainable advancement of the banana industry. Systemic acquired resistance (SAR) mediated by NPR1 (Nonexpressor of Pathogenesis-Related Genes 1) is a key plant defense mechanism, yet the role of banana NPR1 homologs stays unclear. Here, we cloned a NPR1-like gene, *MaNPR11*, from BaXi jiao (BX, *Musa* spp. AAA cv. Cavendish), which was suppressed in susceptible cv. BX but significantly up-regulated in resistant cv. GCTCV-119. Besides, protein-protein interaction assays ascertained that MaNPR11 interacted with 5 MaTGAs in the nucleus. Furthermore, transient overexpression of the *MaNPR11* gene in banana increased the expression levels of immunity-related genes (*MaMAPK*, *MaPAL*, and *MaPR3*), ethylene synthesis-related gene (*MaACO*) and *MaTGA4*, while decreased the level of reactive oxygen species (ROS)-related gene (*MaRBOH*). Notably, overexpression of the *MaNPR11* gene in tobacco significantly strengthened resistance to *Fusarium oxysporum* f. sp. *nicotianae* by promoting the expression of several genes involved in PR and ROS scavenging-related genes. Therefore it is speculated that MaNPR11 may positively regulates banana resistance to *Fusarium oxysporum* f. sp. *cubense* Tropical Race 4 (Foc TR4) by interacting with MaTGA4 and modulating salicylic acid (SA), ethylene (ET), and ROS signaling pathways, serving as a candidate target for disease resistance genetic improvement.

## Introduction

Bananas (*Musa* spp., family *Musaceae*) are perennial herbaceous economic crops, functioning as staple food in tropical regions worldwide. As the world’s most traded and consumed fresh fruit, global net banana imports reached 19 million metric tons in 2024, with fresh banana export value at approximately $15.3 billion (FAO, 2025). They are also a “daily necessity on the dining table” for hundreds of millions people in tropical and subtropical regions (Dale et al., 2017; Wang et al., 2019).

Currently, the most widely commercially cultivated banana variety worldwide is the triploid Cavendish group (*Musa* spp. Cavendish subgroup) (Dale et al., 2017). Asexual propagation is the only propagation method for Cavendish bananas, which results in their low environmental adaptability. The growth and yield of Cavendish bananas are frequently affected by abiotic stresses (drought, low temperature, salinity) and various destructive diseases (Heslop-Harrison and Schwarzacher, 2007; Van Asten et al., 2011; Hu et al., 2017; Ploetz, 2006). Banana fusarium wilt is a soil-borne vascular disease caused by Foc. This pathogen comprises four physiological races: Race 1 (R1), Race 2 (R2), Race 3 (R3), and Race 4 (R4). Among them, R4 is the race with the most severe pathogenicity at present, which can be further divided into the subtropical race 4 (SR4) and tropical race 4 (TR4). TR4 can infect almost all commercial banana varieties, including the globally widely grown Cavendish series, posing a great menace to the world’s banana industry (Ploetz, 2015; Izquierdo-García et al., 2021). Therefore, mining disease-resistant genes and clarifying the disease resistance mechanism in banana are crucial tasks at present.

Systemic acquired resistance (SAR) is an inducible plant defense mechanism that confers broad-spectrum immunity to plants. In the model plant *Arabidopsis thaliana*, AtNPR1 acts as the “master regulator” of SAR, and its functional implementation relies on the precise regulation of SA signaling (Zavaliev and Dong, 2024). Upon pathogen infection or treatment with SA and its functional analogs (e.g., INA, BTH), NPR1 translocates from the cytoplasm to the nucleus and then interacts with the TGA2 transcription factor, thereby activating the expression of pathogenesis-related (PR) genes and ultimately endowing plants with broad-spectrum disease resistance (Wang et al., 2018). In rice, the NPR1 homolog (NH1) retains the interaction mechanism with TGA transcription factors, but its regulatory pattern exhibits distinct species-specificity. *AtNPR1* overexpression efficiently strengthens rice resistance to multiple pathogens, such as *Magnaporthe oryzae* (rice blast fungus) and *Xanthomonas oryzae* pv. *oryzae* (bacterial blight) (Chern et al., 2001; Quilis et al., 2008; Xu et al., 2017). In contrast, overexpression of rice’s endogenous NH1 gene, while improving rice resistance to bacterial blight, leads to hypersensitivity of rice to light and the SA analog BTH. This finding suggests the presence of a unique negative feedback mechanism within the NPR1 regulatory network in rice (Chern et al., 2005; Bai et al., 2011). In wheat, the NPR1 homolog (wNPR1) interacts with 4 TGA and 2 NRR (negative regulators of resistance) homolog proteins. This shares conservation with the mechanism in *Arabidopsis* and rice, where NPR1 regulates PR gene expression through TGA proteins (Cantu et al., 2013).

More and more researches have demonstrated that similar immunity-related mechanisms are present in numerous crops, and regulating the expression profile of NPR1 or its homologous genes can effectively enhance crop disease resistance. For instance, transgenic wheat lines overexpressing *AtNPR1* or *ScNPR1* exhibited enhanced resistance to Fusarium head blight (Makandar et al., 2006; Gao et al., 2013; Yu et al., 2017). Heterologous expression of the *StoNPR1* gene improved the resistance of potato to the fungal pathogen *Verticillium dahliae* (Jue et al., 2014). Overexpression of mulberry *MuNPR1* enhanced the resistance of transgenic *Arabidopsis* to *Pseudomonas syringae* pv. tomato DC3000 (Pst DC3000) (Xu et al., 2019). *AeNPR1a* upregulated the expression levels of PR genes in transgenic tobacco and improved its resistance to bacterial pathogens. Furthermore, the expression of *AeNPR1a* was able to restore the basal resistance of *Arabidopsis* npr1–1 mutants to Pst DC3000 (Sun et al., 2020). Overexpression of *AtNPR1* in the turnip cabbage cultivar Varuna conferred significant resistance to *Sclerotinia sclerotiorum* and *Alternaria brassicae* (Verma et al., 2023). These studies mentioned above revealed the important role of NPR1 in biotic stress and its great application potential in future genetic engineering research.

However, research on the function of banana NPR1 genes is still in its infancy. To date, merely 4 banana NPR1 genes have been successfully cloned and documented in existing literature. These include *MNPR1A* and *MNPR1B* isolated from *Musa* spp. AAA cv. Grand Naine (Endah et al., 2008), *MdNPR1* from *Musa* spp. ABB cv. Dongguan Dajiao (Zhao et al., 2009), and *MuNPR1–1* derived from *Musa* spp. ABB cv. Zhongshan Dajiao (Li et al., 2010). Notably, these prior studies were limited to gene cloning, characterization, and expression profiling, and lacked in-depth exploration of the gene functional roles and underlying regulatory mechanisms. In our previous work, we identified a total of 15 NPR1 genes from the banana A genome, and preliminary findings suggested that *MaNPR11* could serve as a key player in mediating banana resistance against fusarium wilt (Ren et al., 2019). In this study, we successfully cloned the *MaNPR11* gene from BX via homologous cloning. Reverse transcription quantitative PCR (RT-qPCR) was then employed to examine both the tissue-specific expression patterns of this gene and its transcriptional response profiles in fusarium wilt-resistant versus susceptible banana cultivars following Foc TR4 challenge. Our experimental data further revealed that *MaNPR11* exhibits functional similarity to *AtNPR1*. Using the yeast two-hybrid (Y2H) system and bimolecular fluorescence complementation (BiFC) assay, we detected interactions between MaNPR11 and 5 distinct members of the MaTGA transcription factor family. Additionally, when *MaNPR11* was overexpressed in tobacco plants, a notable enhancement in resistance to tobacco fusarium wilt was observed. On the basis of these results, we hypothesize that *MaNPR11* exerts a critical function in modulating banana resistance to fusarium wilt.

## Materials and methods

### Plant materials, treatments and expression pattern analysis

The Cavendish banana exhibits high susceptibility to TR4. In contrast, Giant Cavendish Tissue Culture Variants (GCTCV) have developed resistance to TR4 via tissue culture-induced variation (Hwang and Ko, 2004), among which GCTCV-119 stands out as the most optimal TR4-resistant replacement cultivar for Cavendish (Ploetz, 2015). The two banana cultivars used in the study were BX and GCTCV-119. For tissue expression analysis, samples of roots, rhizomes, pseudostems, leaves, flowers, and fruits were collected from BX. For Foc TR4 stress, the roots of 2-month-old cultivars BX and GCTCV-119 were dipped in Foc TR4 spore suspension of 1.0×10^6^ spores/mL, and then harvested the roots at 0, 2, 4, and 6 days post-infection (DPI) (Wang et al., 2012).

Total RNA was obtained via the RNA extraction kit (DP432, Tiangen, Beijing, China). First-strand cDNA was synthesized according to the protocol provided with the Revert Aid First Strand cDNA Synthesis Kit (K1622, Thermo Fisher, Massachusetts, USA). The coding sequence (CDS) of *MaNPR11* was cloned from BX. Gene expression was detected according to the protocol provided with the ChamQ Blue Universal SYBR qPCR Master Mix (Q312, Vazyme, Nanjing, China). *MaActin* served as an internal control for normalizing the expression levels of the target genes. The resulting data were analyzed using the 2^-ΔΔCT^ method (Livark and Schmittgen, 2001) and visualized with GraphPad Prism 6.0 software. All primers used in the study are presented in **Supplementary Table 1**, with the experiments conducted using three biological replicates and three technical replicates.

### Yeast two-hybrid assay

Y2H assay was executed following the manufacturer’s instructions (Clontech, SF, USA). The CDS of *MaNPR11* was ligated into the pGBKT7 vector to create a bait construct (BD). The CDS of *MaTGA1*, *MaTGA2*, *MaTGA4*, *MaTGA6*, *MaTGA7*, *MaTGA8*, and *MaTGA9* were ligated into the pGADT7 vector to create prey constructs (AD), respectively. pGBKT7-MaNPR11 and the empty pGBKT7 vector were individually introduced into yeast AH109, plated on SD/-Trp, SD/-Trp/X-α-gal, and SD/-Trp/-His/X-α-gal solid media at 30 °C for 3 d. Fusion protein toxicity and self-activation were assessed by yeast colony growth. For interaction testing, AD/BD plasmid combinations were co-transformed into AH109 and plated onto SD/-Trp/-Leu, SD/-Trp/-Leu/-His/-Ade, and SD/-Trp/-Leu/-His/-Ade/X-α-Gal solid media at 30 °C for 3 d. Co-transformation of pGADT7-T and pGBKT7–53 was the positive control, and co-transformation of pGADT7-T and pGBKT7-Lam was the negative control.

### Bimolecular fluorescence complementation assay

The CDS of *MaNPR11* was ligated into the pNC-BiFC-ECC vector, and the CDSs of *MaTGA1*, *MaTGA2*, *MaTGA4*, *MaTGA8*, and *MaTGA9* were separately cloned into the pNC-BiFC-ENC vector via Nimble Cloning (Yan et al., 2023). The interaction between papaya eIFiso4E and prsv-Vpg located in the nucleus was used as the positive control (Yan et al., 2023). All constructs and control vector were individually introduced into *Agrobacterium tumefaciens* GV3101 (pSoup-p19). MaNPR11-pNC-BiFC-ECC and each MaTGAs-pNC-BiFC-ENC *Agrobacterium* cultures were mixed at 1:1 and infiltrated into tobacco leaves. Fluorescence signals were examined by confocal laser scanning microscopy (Olympus, FluoView FV1000, Tokyo, Japan) at 72 hours post-inoculation (HPI).

### Transient transformation of banana roots

The construction of pCAMBIA3300-*MaNPR11* was transformed into *Agrobacterium tumefaciens* strain GV3101. Banana seedlings at the 5-leaf-1-heart growth stage were selected for experiments. Their root systems were thoroughly washed, patted dry with paper towels, and sectioned into fragments of roughly 1 cm in length. To inhibit root browning, the root fragments were immersed in a 0.5% sodium bisulfite solution. Following the pretreatment, the root fragments were then incubated in *Agrobacterium tumefaciens* suspension harboring pCAMBIA3300-*MaNPR11* for a 1-hour infection period. Excess bacterial suspension on the root fragments was removed by blotting with filter paper, and the roots were then placed onto MS solid medium supplemented with 100 μmol/L acetosyringone (AS). After 24 hours of dark incubation at 26 °C, samples were harvested and defined as the 0 HPI. Roots that had undergone 24-hour *A. tumefaciens* infection were further treated with a Foc TR4 spore suspension (with a spore density of 1.0×10^6^ spores/mL) for 1 hour. Additional sampling was conducted at 6 and 12 HPI. The empty vector served as a control (Mock). All harvested samples were instantly snap-frozen in liquid nitrogen and stored at -80 °C until further gene expression analysis.

### Tobacco genetic transformation and the evaluation of its disease resistance

pCAMBIA3300-*MaNPR11* was transformed into *Nicotiana benthamiana* via the leaf-disk method (Sparkes et al., 2006). Herbicide-based screening of the T3 generation yielded transgenic plants, which were further verified by DNA, RNA, and bar protein analyses. Transgenic and wild-type (WT) tobacco roots were inoculated with *Fusarium oxysporum* f. sp. *nicotianae* spore suspension (1×10^6^ spores/mL) via root irrigation. After 2 h, plants were cultured at 30 °C with 90% relative humidity. Tobacco leaves at 0 and 24 HPI were collected for PR and ROS-scavenging genes expression analysis. The primer sequences are provided in **Supplementary Table 1**. Phenotypes were recorded continuously, with photos taken at 14 DPI. For cell viability assessment, transgenic and WT tobacco leaves were immersed in *Fusarium oxysporum* f. sp. *nicotianae* spore suspension (1×10^6^ spores/mL) for 2 h, followed by trypan blue staining to detect cell death. The staining area was then calculated by ImageJ 1.53 software.

## Results

### Cloning and expression of MaNPR11

**Figure 1:**
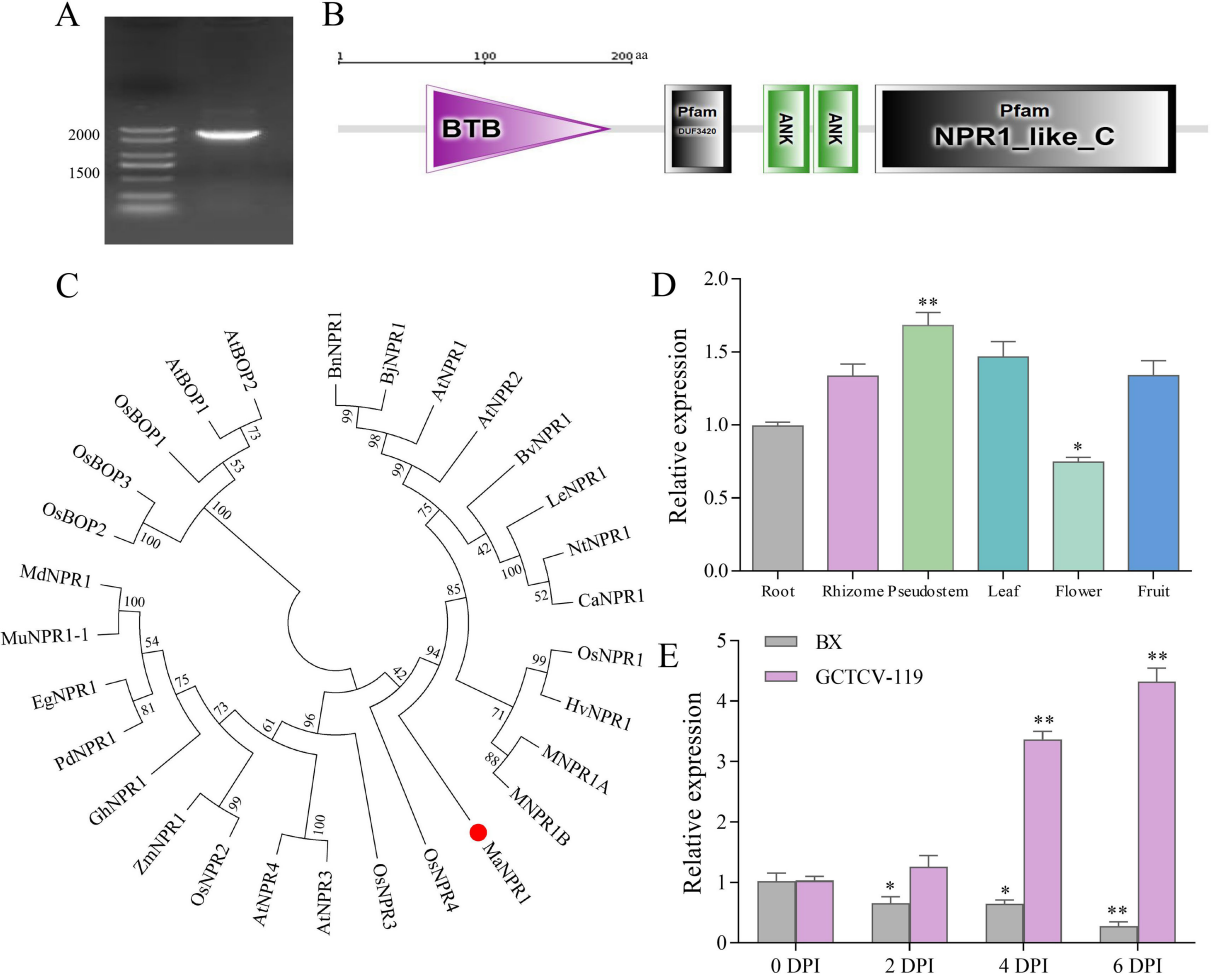
Characterization of *MaNPR11* in banana. **(A)** Agarose gel electrophoresis of PCR product of *MaNPR11* gene. **(B)** Protein domains of MaNPR11. **(C)** Phylogenetic analysis of NPR1 from different plant species. The informations of the other NPR1 proteins were listed as follows. *Arabidopsis thaliana* (AtNPR1, NP_176610; AtNPR2, NP_194342; AtNPR3, NP_199324; AtNPR4, NP_193701; AtBOP1, NP_001190116; AtBOP2, NP_181668); *Brassica juncea* (BjNPR1, ABC94642.2); *Brassica napus* (BnNPR1, AAM88865.2); *Beta vulgaris* (BvNPR1, AY640381.1); *Capsicum annum* (CaNPR1, ABG38308.1); *Elaeis guineensis* (EgNPR1, XP_010908601.1); *Gladiolus hybrid* (GhNPR1, KJ769203); *Hordeum vulgare* (HvNPR1, AM050559.1); *Lycopersicon esculentum* (LeNPR1, AY640378.1); *Musa* spp. ABB cv. Zhongshang Dajiao (MuNPR1-1, ACE86413.1); *Musa* spp. ABB cv. Dongguan Dajiao (MdNPR1 ACJ04031.1); *Musa* spp. AAA cv. Grand Naine (MNPR1A, DQ925843.1; MNPR1B, EF137717.1); *Nicotiana tabacum* (NtNPR1, AAM62410.1); *Oryza sativa* (OsNPR1, LOC_Os01g09800.1; OsNPR2, LOC_Os01g56200.1; OsNPR3, LOC_Os03g46440.1; OsNPR4, LOC_Os01g61990.2; OsBOP1, LOC_Os01g72020.1; OsBOP2, LOC_Os11g04600.1; OsBOP3, LOC_Os12g04410.1); *Phoenix dactylifera* (PdNPR1, XP_008806697.3); *Zea mays* (ZmNPR1, NP_001147587.1). **(D, E)** Expression analysis of *MaNPR11* in various tissues and in response to Foc TR4 inoculation. Banana cultivar GCTCV-119 was Foc-TR4-resistant, while BX was susceptible. Data were the mean ± SE of three separate biological replicates. Statistical differences among samples were assessed by Student’s t test (*P < 0.05; **P < 0.01).

The full-length cDNA of *MaNPR11* was cloned from BX. It was 1746 bp in length and encoded a 581 amino acid protein of 64.18 kDa, and the hypothetical isoelectric point (pI) was 5.70 (**Figure 1A**). The putative MaNPR11 protein had typical conserved domains of the NPR1 family, including a BTB/POZ domain (broadcomplex, Tramtrack and Bric-a-brac/poxvirus and zinc finger protein domain), an ankyrin repeat domain that mediates interaction with TGA transcription factors, and a NPR1-like C-terminal region (transactivation domain). And it also had a DUF domain with unknown function (**Figure 1B**). Sequence alignment was performed between *MaNPR11* and four previously reported NPR1 genes in banana (*MNPR1A*, *MNPR1B*, *MdNPR1*, and *MuNPR1-1*). The percentage identity between MaNPR11 and these four sequences was 51.12%, 54.92%, 40.84%, and 40.14%, respectively. Phylogenetic analysis showed the evolutionary relationship between *MaNPR11* and NPR1-like genes in *Musa* and other plants (**Figure 1C**).

The expression of *MaNPR11* was constitutively expressed in banana various tissues, with the highest expression in the pseudostem, followed by the leaf and fruit, and the lowest in the flower (**Figure 1D**). In resistant and susceptible varieties of banana after inoculation with Foc TR4 for 0, 2, 4, 6 DPI, the expression of *MaNPR11* gradually decreased after inoculation in the susceptible variety, reaching the lowest level at 6 DPI. While in the resistant variety, *MaNPR11* expression significantly increased, reaching the maximum at 6 DPI, with 4.2 times that of the control (**Figure 1E**). It can be seen that the transcripts of *MaNPR11* was suppressed in the susceptible variety and activated in the resistant variety. These findings indicate that *MaNPR11* is likely to serve as a key regulator in mediating banana’s defense response to Foc TR4.

### MaNPR11 could interact with MaTGAs

It is reported that NPR1 positively regulates systemic acquired resistance by inducing the expression of PR genes through interacting with TGA transcription factors. To validate the interaction between MaNPR11 and MaTGAs, we previously identified 9 *MaTGAs* from the banana A genome, named *MaTGA1*-*MaTGA9*. Subsequently, we successfully cloned the CDS of 7 *MaTGAs* (*MaTGA1*, *MaTGA2*, *MaTGA4*, *MaTGA6*, *MaTGA7*, *MaTGA8*, and *MaTGA9*) from BX, and then conducted a Y2H-based interaction screening to identify their binding relationships with MaNPR11.

**Figure 2:**
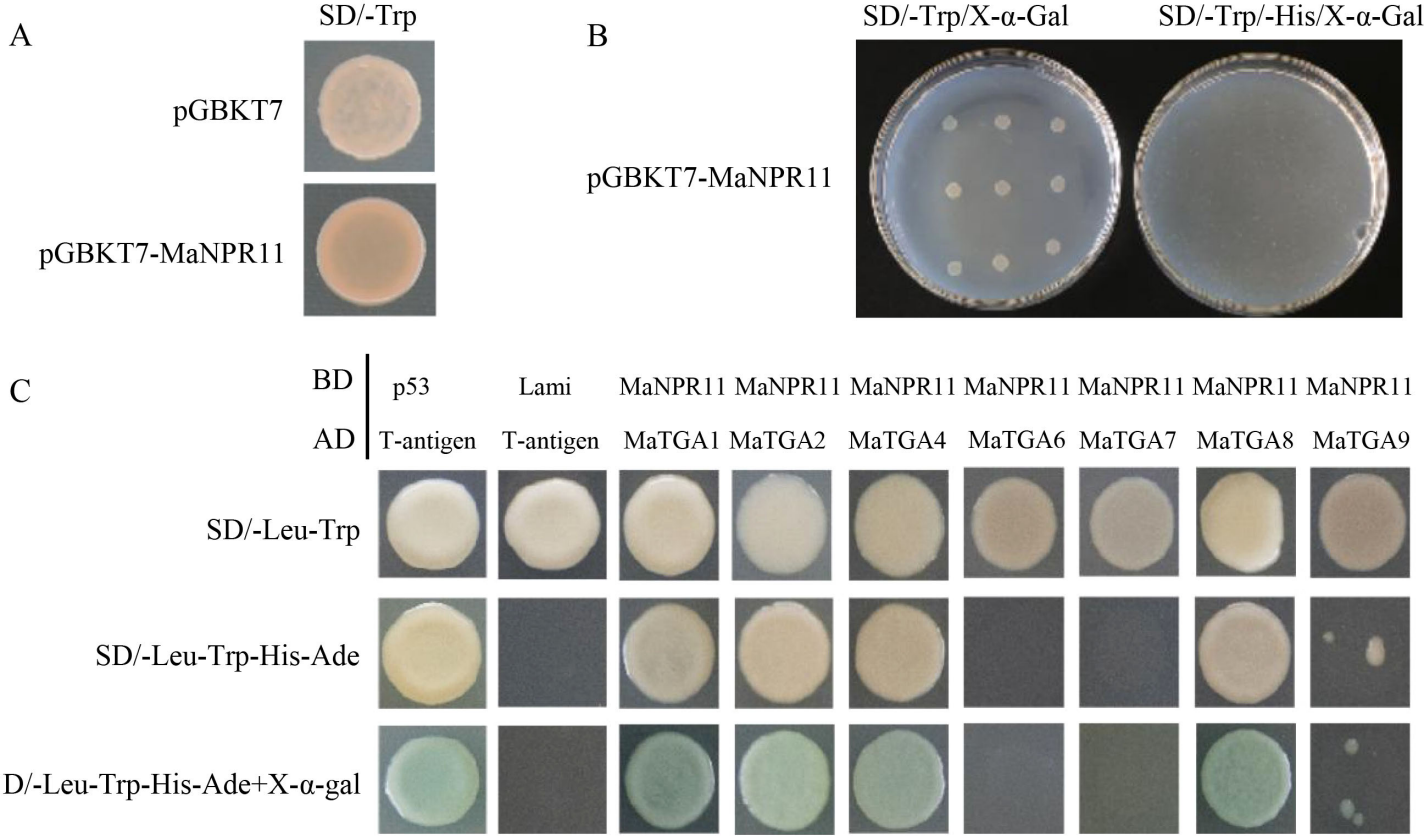
Y2H analysis of interactions between MaTGAs and MaNPR11. **(A, B)** The toxicity and auto-activation assays for the bait vector. **(C)** Y2H verification of the interaction between MaTGAs and MaNPR11. The interaction was examined in the SD/-Leu-Trp-His-Ade medium containing X-α-gal.

The toxicity and auto-activation assays were tested for the bait vector (**Figures 2A, B**). Except for MaTGA6-MaNPR11 and MaTGA7-MaNPR11, the yeast cells containing MaTGA1-MaNPR11, MaTGA2-MaNPR11, MaTGA4-MaNPR11, MaTGA8-MaNPR11, MaTGA9-MaNPR11 and the positive control grew well and showed a blue color on SD/-Trp/-His/X-α-Gal medium, indicating the interaction between MaTGAs and MaNPR11. Among them, the interaction intensity between MaTGA9 and MaNPR11 was relatively weaker than others (**Figure 2C**).

**Figure 3:**
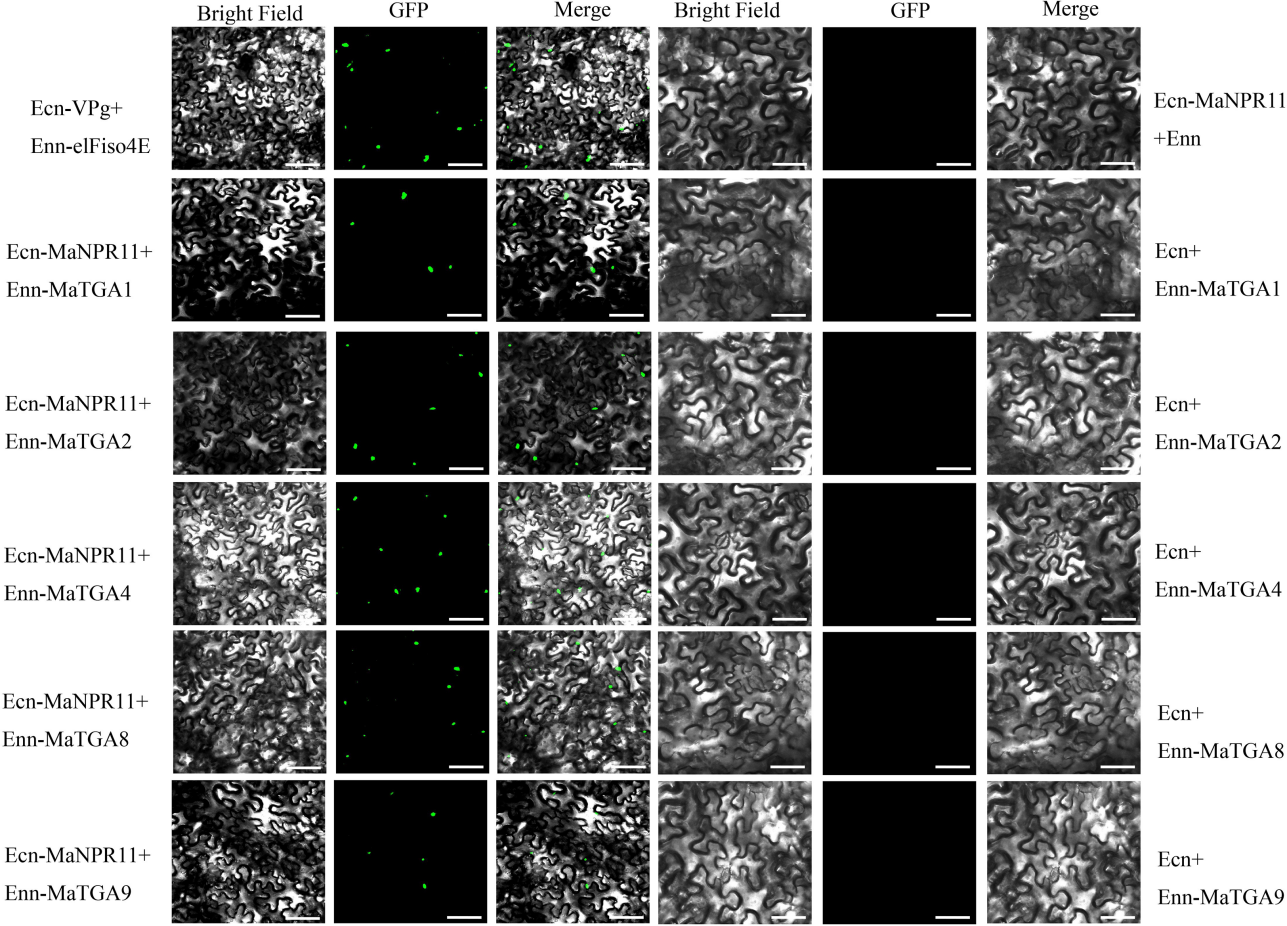
BiFC assay verified the interaction between MaTGA1, MaTGA2, MaTGA4, MaTGA8, MaTGA9 and MaNPR11. The interaction between papaya eIFiso4E and prsv-Vpg located in the nucleus was used as the positive control. Scale bars, 50μm.

The interaction between MaTGAs and MaNPR11 was further verified via the BiFC assay. Tobacco cells that harbored MaTGA1-MaNPR11, MaTGA2-MaNPR11, MaTGA4-MaNPR11, MaTGA8-MaNPR11, and MaTGA9-MaNPR11 showed a green color and all these signals were localized in the cell nucleus. In contrast, no green fluorescent signals were detected in tobacco cells harboring the negative control plasmids (**Figure 3**).

### Transient overexpression of *MaNPR11* in banana roots influenced the transcripts of ROS-, ET-, immunity-related genes, and MaTGAs interacting with MaNPR11

**Figure 4:**
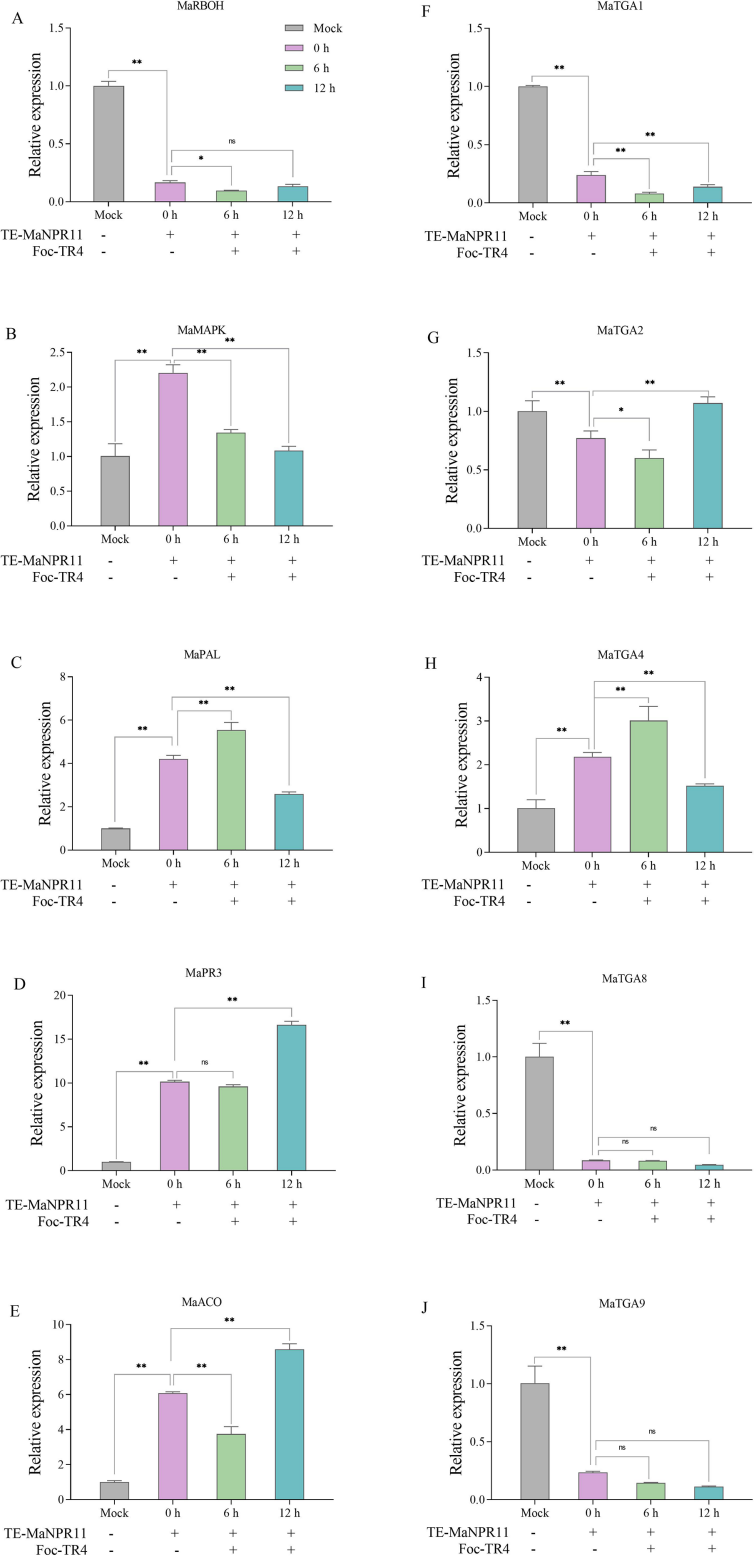
Transient overexpression of *MaNPR11* in banana roots influenced the transcripts of ROS-, ET-, immunity-related genes, and MaTGAs interacting with MaNPR11. **(A)** The expression levels of one ROS-related gene (*MaRBOH*). **(B–D)** The expression levels of three immunity-related genes (*MaMAPK, MaPAL*, and *MaPR3*). **(E)** The expression levels of one ET-related gene (*MaACO*). **(F–J)** The expression levels of 5 MaTGAs interacting with MaNPR11 (*MaTGA1*, *MaTGA2*, *MaTGA4*, *MaTGA8*, and *MaTGA9*). Mock, empty vector. Data were the mean ± SE of three separate biological replicates. Statistical differences among samples were assessed by Student’s t test (*P < 0.05; **P < 0.01).

After Foc TR4 inoculation in *MaNPR11*-overexpressing (*MaNPR11*-OE) roots, the transcripts of ROS-, ET-, and immunity-related genes, as well as MaTGAs interacting with MaNPR11 proteins, were analyzed. *MaRBOH*, a key gene involved in ROS production, showed decreased expression (**Figure 4A**). We speculate that *MaNPR11* may maintain low ROS levels by suppressing the expression of *MaRBOH*, thereby ensuring normal plant growth and development. The expression levels of three immunity-related genes (*MaMAPK*, *MaPAL*, and *MaPR3*) and one ethylene synthesis-related gene (*MaACO*) were significantly up-regulated compared to the mock (**Figures 4B–E**). Moreover, after Foc TR4 inoculation, the expression levels of *MaPAL* and *MaPR3* were significantly up-regulated at 6 and 12 HPI, respectively, indicating the activation of downstream signaling pathways (**Figures 4C, D**). Notably, the expression of *MaACO* was significantly up-regulated at 12 HPI (**Figure 4E**). These results imply that, in addition to SA, ET may also play a positive role in pathogen resistance in banana.

Regarding the expression of *MaTGA*s, compared to the mock, the expression levels of *MaTGA1*, *MaTGA2*, *MaTGA8*, and *MaTGA9* were remarkably down-regulated, whereas that of *MaTGA4* was significantly up-regulated (**Figures 4F–J**). After Foc TR4 inoculation, the expression levels of *MaTGA1*, *MaTGA8*, and *MaTGA9* still remained down-regulated (**Figures 4F, I**, J), while that of *MaTGA2* showed a trend of initial decline followed by a slight increase (**Figure 4G**). In contrast, the expression of *MaTGA4* was up-regulated by 2.3-fold at 6 HPI compared to its expression level at 0 HPI (**Figure 4H**). These findings indicate that *MaTGA4* is positively regulated by *MaNPR11* and may play an active role in mediating disease resistance.

### *MaNPR11* overexpression significantly induces resistance to *Fusarium oxysporum* f. sp. *nicotianae*

**Figure 5:**
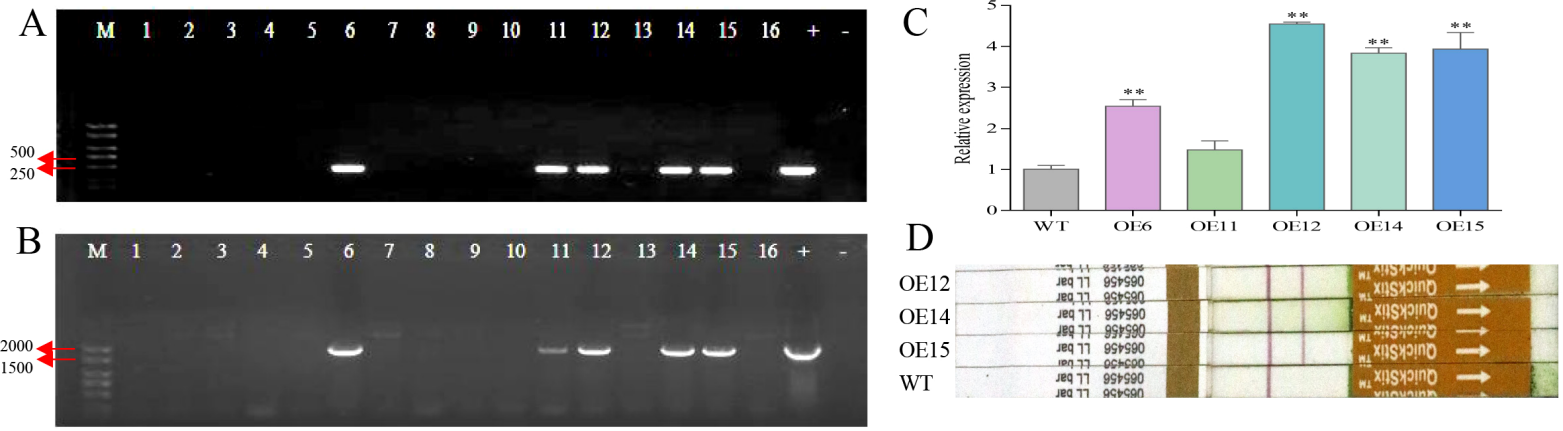
Identification of transgenic lines in tobacco. **(A, B)** PCR amplification of herbicide gene (Phosphinothricin) and *MaNPR11* in transformed tobacco lines. M, DNA marker; 1-16, transgenic tobacco plants; +, positive control; -, negative control. **(C)** qRT-PCR analysis of the expression levels of *MaNPR11* in different transgenic lines. Data are means ± SD calculated from three biological experiments. Statistical significance was examined using Student’s *t* test (*P < 0.05; **P < 0.01). Three overexpressing *MaNPR11* transgenic lines (OE12, OE14 and OE15) with high transcript level were selected for further experiment. **(D)** Detection of the bar protein in three selected positive plants.

To study the function of *MaNPR11* during pathogen infection, the over-expression vector pCAMBIA3300-*MaNPR11* was genetically transformed into *N. benthamiana*. Totally, 5 transgenic tobacco plants over-expressing *MaNPR11* were obtained by testing at the DNA level and transcriptional level (**Figures 5A–C**). Three transgenic lines (OE12, OE14, and OE15) with high expression level were further tested at bar protein level (**Figure 5D**) and selected for inoculation with *Fusarium oxysporum* f. sp. *nicotianae*.

**Figure 6:**
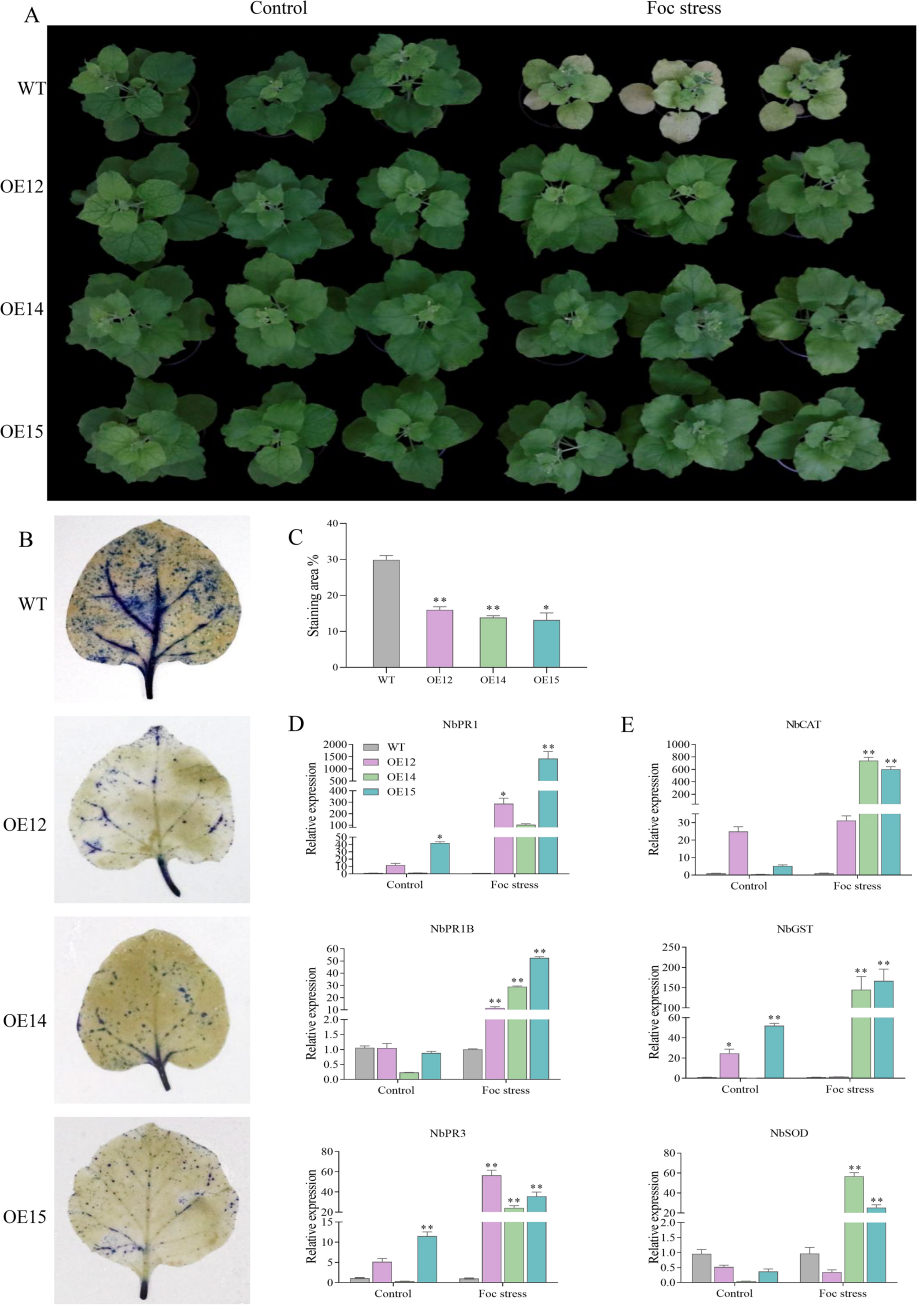
Disease resistance of *MaNPR11* overexpressing transgenic tobacco. **(A)** Disease phenotypes of *MaNPR11* transgenic lines after infecting with *Fusarium oxysporum* (f) sp. *nicotianae* for 14 days. **(B)** Trypan blue staining of tobacco leaves in *MaNPR11* transgenic and wild-type lines after immersion inoculation with *Fusarium oxysporum* (f) sp. *nicotianae*. **(C)** Quantification of trypan blue staining area after inoculation with *Fusarium oxysporum* (f) sp. *nicotianae*. **(D, E)** The expression levels of PR and ROS scavenging-related genes. Data were the mean ± SE of three separate biological replicates. Statistical differences among samples were assessed by Student’s t test (*P < 0.05; **P < 0.01).

After inoculation with *Fusarium oxysporum* f. sp. *nicotianae* 14 d, the WT tobacco leaves exhibited obvious yellowing and the plants showed significant growth inhibition. In contrast, the *MaNPR11* transgenic lines maintained a good growth status, with their leaves remaining green and no obvious disease symptoms observed (**Figure 6A**). Following immersion inoculation with the spore suspension of *Fusarium oxysporum* f. sp. *nicotianae* for 2h, trypan blue staining of tobacco leaves in WT exhibited intense blue precipitation around veins, with stained regions merging into patches, indicating extensive and severe cell death. Conversely, only scattered and faint blue spots were detected at the leaf margins or occasional damaged sites in *MaNPR11*-OE leaves, with most mesophyll cells remaining unstained, suggesting a significantly lower level of cell death and intact cell membrane integrity (**Figures 6B, C**). Additionally, the transcripts of PR and ROS-scavenging genes were similarly up-regulated in the transgenic plants following pathogen challenge (**Figures 6D, E**). These findings indicated that the stable overexpression of the *MaNPR11* gene could enhance the disease resistance of *N. benthamiana* to pathogen infection by promoting the expression of PR and ROS scavenging-related genes.

## Discussion

Banana is one of the most widely cultivated and economically valuable cash crops in tropical regions worldwide (Arshad et al., 2025). However, the vast majority of commercially grown Cavendish cultivars are susceptible to various pathogens, particularly Foc TR4, leading to substantial economic losses (Ploetz, 2015; Izquierdo-García et al., 2021; Chen et al., 2023). To date, no effective control measures have been established. Given this challenge, deciphering the mechanisms regulating banana defense responses holds profound significance for banana breeding programs, as it will facilitate the screening of germplasm resources with novel disease resistance traits.

In the study, we focused on the NPR1 gene, a well-documented key regulator of plant disease resistance (Cao et al., 1997; Zavaliev and Dong, 2024). NPR1 not only directly participates in the regulation of plant SAR and other types of induced disease resistance but also plays a core regulatory role in gene-mediated plant disease resistance responses and basal defense responses (Cao et al., 1997; Durrant and Dong, 2004). We isolated an NPR1-like gene (*MaNPR11*) from BX and analyzed its role in regulating tobacco tolerance to *Fusarium oxysporum* f. sp. *nicotianae*. Bioinformatic analysis revealed that the amino acid sequence has a molecular weight of 64.18 kDa and an isoelectric point of 5.70, and contains complete BTB/POZ domains, ankyrin repeat (ANK) sequences, and an NPR1-like-C domain (**Figure 1B**). It is consistent with the characteristics of previously reported NPR1 proteins from other plant species (Cao et al., 1997; Rochon et al., 2006; Endah et al., 2008; Zhao et al., 2009; Li et al., 2010; Wang et al., 2017). Additionally, a phylogenetic tree showed that MaNPR11 clustered with its orthologous proteins from *Arabidopsis thaliana* and other plants with high bootstrap support (**Figure 1C**), indicating evolutionary conservation and suggesting potential conserved functions between homologous proteins in banana and *Arabidopsis*.

To further explore *MaNPR11* role in disease resistance, its cultivar-specific expression pattern in response to Foc TR4 was analyzed.

The results showed that *MaNPR11* exhibits divergent expression trends in resistant versus susceptible banana varieties (**Figure 1E**). Similar results were also found in *MNPR1A*, *MuNPR1-1*, and pepper *CaNPR* genes (Endah et al., 2008; Zhao et al., 2009; Ishfaqe et al., 2024). These results demonstrate that *MaNPR11* expression is suppressed in susceptible cultivars but significantly activated in resistant/tolerant cultivars, implying that *MaNPR11* may play a crucial role in regulating banana fusarium wilt resistance.

NPR1 and TGAs’ interaction is essential for NPR1 function in *Arabidopsis* and rice (Silva et al., 2018). We also evaluated the potential interactions between MaNPR11 and 7 MaTGAs using Y2H and BiFC assay. Strong interactions were detected between MaNPR11 and MaTGA1, MaTGA2, MaTGA4, and MaTGA8 respectively, while the interaction between MaNPR11 and MaTGA9 was weak (**Figure 2C**). Similar NPR1-TGAs interaction patterns have been reported in plants such as *Arabidopsis* (Fan and Dong, 2002), rice (Chern et al., 2001), wheat (Cantu et al., 2013), *Gladiolus hybridus* (Zhong et al., 2015), and sand pear (Xu et al., 2025). It was also supported by Lin et al., 2021. They found that MaTGA8 had strong interaction with MaNPR11 or weaker interaction with MaNPR4 (Lin et al., 2021).

To link these interactions to downstream defense responses, *MaNPR11* was transiently expressed in BX roots via the *Agrobacterium*-mediated method, followed by inoculation with Foc TR4. During this process, the transcripts of ROS-, ET-, and immunity-related marker genes, as well as MaTGAs that interact with MaNPR11, were analyzed. RBOH gene, participating in production of ROS, was found to be up-regulated in BX at 5 and 10 DPI. While in the resistant cv. Yueyoukang 1, RBOH expression level was suppressed by TR4 at 0.5, 1 and 3 DPI and had no any change at 5 and 10 DPI (Bai et al., 2013). It was been reported that germinating TR4 spores were observed to colonize BX roots at 5 DPI (Li et al., 2011). They guessed RBOH was induced by TR4 in BX to produce hypersensitive reaction (HR) which may benefit TR4 further infection (Bai et al., 2013). The same results were also found in Citrus. CsRboh06 showed opposite expression trends in response to *Xanthomonas citri* subsp. *citri* (Xcc). The expression level of CsRboh06 was gradually upregulated in the susceptible cultivar Wanjincheng, while gradually downregulated in the resistant cultivar Sijiju within 24 HPI (Qin et al., 2020). In our study, RBOH expression level decreased in *MaNPR11*-OE BX roots and was also suppressed by TR4 at 6 and 12 HPI (**Figure 4A**). Compared with the resistant cv. Yueyoukang 1, the same expression trend of RBOH gene was observed in *MaNPR11*-OE BX roots. Potentially, *MaNPR11* may suppress TR4 further invasion by negatively regulating RBOH to block HR in *MaNPR11*-OE BX roots. However, this hypothesis needs to be further examined. The increased expression of *MaPAL*, *MaPR3*, and *MaACO* was observed in *MaNPR11*-OE roots under TR4 stress (**Figures 4C–E**), suggesting the downstream signaling pathways were activated.

As for *MaTGA*s, only *MaTGA4* was significantly upregulated and peaked at 6 HPI (**Figure 4H**). Compared with mock control, the expression of *MaTGA1*, *MaTGA8*, and *MaTGA9* was significantly suppressed (**Figures 4F, I, J**). Under TR4 stress, the decreased expression of *MaTGA1*, *MaTGA8*, and *MaTGA9* was observed, suggesting distinct response patterns in *MaNPR11*-OE roots. However, whether the MaNPR11-MaTGA interactions trigger the banana SAR response by regulating PR gene transcription remains to be further verified. Future research should focus on verifying its function in stably transgenic bananas, elucidating the precise mechanism of PR gene regulation mediated by the MaNPR11-MaTGA complex, and exploring its interaction with other defense signaling components to fully dissect the banana fusarium wilt resistance network.

Additionally, transgenic tobacco plants with stable *MaNPR11* overexpression exhibited significant upregulation of PR genes and enhanced resistance to tobacco fusarium wilt. Specifically, under pathogen stress, WT tobacco leaves showed obvious chlorosis and significant growth inhibition, whereas the transgenic lines maintained good growth status without obvious leaf chlorosis (**Figure 6A**). Trypan blue staining revealed a significant reduction in leaf cell death in the transgenic lines (**Figures 6B, C**). The transcripts of PR and ROS scavenging-related genes were increased in *MaNPR11-*OE lines (**Figures 6D, E**), indicating that downstream signaling pathways were activated, thereby promoting the biosynthesis of defense-related factors. These findings support *MaNPR11* as a promising target for enhancing disease resistance in banana.

## Conclusion

In this study, we cloned and identified *MaNPR11* from BX. The protein contains conserved NPR1 domains and is evolutionarily conserved with homologous genes from other plants. Its expression was activated in resistant cultivars but suppressed in susceptible cultivars after Foc TR4 infection. Functional verification showed that MaNPR11 interacted with 5 MaTGA transcription factors in the nucleus, among which MaTGA4 was a key positive downstream regulator. Transient overexpression of *MaNPR11* in banana roots regulated the expression of ROS-, ET-, and immunity-related genes. Stable overexpression of *MaNPR11* in tobacco enhanced its resistance to fusarium wilt, characterized by reduced disease symptoms, decreased cell death, and upregulated defense genes. This study confirms that MaNPR11 regulates banana resistance to Foc TR4 by interacting with MaTGAs and coordinating SA, ET, and ROS signaling networks, making it an excellent candidate gene for disease resistance genetic improvement.

## Data Availability

The original contributions presented in the study are included in the article and **Supplementary Material**. Further inquiries can be directed to the corresponding author.
